# Triple-Rod Construct Approach for Severe Rigid Scoliosis: A Comprehensive Case Series

**DOI:** 10.7759/cureus.81546

**Published:** 2025-03-31

**Authors:** Thirumurugan Kurusamy, Mohamed Faizal B Abdul Manan, Dzulkarnain Amir, Fazir Mohamad

**Affiliations:** 1 Department of Orthopedic Surgery, General Hospital Kuala Lumpur, Kuala Lumpur, MYS

**Keywords:** apical region correction, global spinal realignment, rigid scoliosis, spinal deformity correction, triple-rod technique

## Abstract

Introduction and aim: Scoliosis is a complex three-dimensional deformity of the spine that leads to lateral curvature, rotation, and imbalance. The severity of scoliosis varies, ranging from mild cases requiring observation to severe, rigid deformities that may necessitate surgical intervention. The surgical management of severe rigid scoliosis carries with it several devastating complications, such as neurological injury, bleeding, implant failure, or loss of correction. The triple-rod technique is an advanced spinal instrumentation method for correcting severe and rigid scoliosis, involving the sequential placement of three rods to enhance deformity correction, reduce mechanical stress on the primary rods, and improve the stability of the construct. These are eight patients among many who presented with varying etiologies of severe rigid scoliosis, but were all treated with a triple-rod posterior instrumentation approach.

This case series aimed to evaluate the surgical outcomes of the triple-rod technique in patients with severe and rigid scoliosis from various etiologies, including idiopathic, congenital, neuromuscular, and syndromic scoliosis. This study examines the surgical outcomes of the triple-rod technique by evaluating the extent of coronal and sagittal correction achieved, assessing the efficacy of correction in the primary and secondary structural curves of rigid scoliosis, and documenting any intra-operative or post-operative complications.

Method: This retrospective single-center study analyzed patients with severe and rigid scoliosis treated surgically using the triple-rod technique. Inclusion criteria included a main coronal curve of >90° and a flexibility index of <25%, excluding those with prior traction or spinal surgery. Pre-operative and post-operative radiographs were used to measure curve angles and assess surgical correction. Post-operatively, all patients were monitored for complications, including neurological function, intra-operative neuromonitoring changes, wound infections, and thromboembolic events. Neurological assessments were conducted at regular intervals, evaluating muscle strength, deep tendon reflexes, and sensory responses. Post-operative radiographs were obtained to assess implant positioning, hardware-related issues, curve correction maintenance, and overall spinal alignment.

Result: Eight patients with severe rigid scoliosis underwent surgical correction using the triple-rod technique. The mean age at surgery was 16.3 years (range: 13-24 years). The major coronal Cobb angle improved significantly from a pre-operative mean of 97.9°±7.1° to 51.6°±10.9° post-operatively, while the sagittal Cobb angle improved from 53.9°±23.5° to 35.6°±9.5°. The triple-rod technique demonstrated significant correction, with the main thoracic curve showing the greatest improvement. All cases were completed without complications, including neurological deficits, intra-operative monitoring changes, infections, thromboembolic events, or hardware-related issues.

Conclusion: The triple-rod technique offers an effective solution, providing substantial correction of coronal and sagittal deformities associated with severe rigid scoliosis. Its primary advantage is the ability to achieve a gradual and controlled correction of spinal deformity. This technique helps minimize the risk of neurological complications and other surgical morbidities associated with severe rigid scoliosis surgery.

## Introduction

Scoliosis is a complex three-dimensional deformity of the spine that leads to curvature, rotation, and imbalance [1]. While the most common form is adolescent idiopathic scoliosis, other etiologies such as congenital, neuromuscular, and syndromic conditions can also result in severe and rigid spinal deformities [2]. The severity of scoliosis varies, ranging from mild cases requiring observation to severe, rigid deformities that may necessitate surgical intervention to prevent progression and associated complications. Severe rigid scoliosis is characterized by a primary coronal curve exceeding 90° in magnitude. The treatment of severe and rigid scoliosis is challenging, where severe scoliosis patients are first seen in a spine centre after years neglected deformity progression, presenting with large curves, severe thoracic hump, shoulder and trunk imbalance. In severe scoliosis cases, the spine may be significantly rotated and curved, which can pose challenges during surgical intervention [3]. Some potential risks associated with surgery for severe scoliosis include neurological injury during surgical reduction of curve, bleeding, implant failure, and extended recovery time [4]. The triple-rod technique is an advanced spinal instrumentation method used in the surgical correction of severe and rigid scoliosis. This technique involves the sequential placement of three rods to optimize the correction of structural spinal curves, distribute mechanical loads more effectively, and minimize stress on the primary rods. By reinforcing the construct, the additional rod enhances overall spinal stability, reduces the risk of implant failure, and promotes a more balanced post-operative alignment. This technique is particularly beneficial in cases requiring significant correction of coronal and sagittal imbalance, where traditional dual-rod constructs may be insufficient. This case series highlights eight patients with various underlying conditions who presented with severe rigid scoliosis and underwent successful surgical correction using the triple-rod technique, demonstrating its effectiveness and safety in managing complex spinal deformities.

## Materials and methods

Methodology

This was a retrospective analysis of a single-center case series, in which patients with severe and rigid scoliosis were surgically treated using the triple-rod technique. All procedures were performed by the same senior spinal surgeon at our institution between July 2023 and December 2024. The inclusion criteria were as follows: patients with severe scoliosis defined as a main coronal curve of >90° and rigid scoliosis defined by a flexibility index of <25%. Patients were excluded if they had undergone prior spinal surgery. Whole spine erect anteroposterior and lateral radiographs were obtained before surgery. Supine side bending radiographs were also obtained to evaluate curve flexibility.

Pre-operative measurements included the main thoracic Cobb angle, minor compensatory coronal Cobb angle, and major sagittal Cobb angle. All patients included in this study underwent surgical treatment involving apical region correction and global balance using a three-rod construct.

Post-operatively, all patients were closely monitored for potential complications, including neurological function, intra-operative neuromonitoring changes, and other surgery-related issues. Neurological assessments were performed at regular intervals, including immediate post-operative evaluations in the recovery unit and subsequent follow-ups during hospitalization and outpatient visits. Motor and sensory functions were assessed using standardized neurological examination protocols, including the evaluation of muscle strength, deep tendon reflexes, and sensory responses in all extremities.

All patients were also evaluated for other post-operative complications, including wound infections and pulmonary or thromboembolic events. Post-operative radiographs were routinely obtained to assess implant positioning, hardware-related issues (such as implant loosening, rod breakage, or screw pullout), curve correction maintenance, and overall spinal alignment.

The data were analyzed using IBM SPSS Statistics for Windows version 25.0 (Armonk, NY: IBM Corp.), released 2017. Data analysis was done using paired-sample t-test to compare pre-operative and post-operative Cobb angles for each of the curve types (proximal thoracic, main thoracic, thoracolumbar/lumbar, and kyphosis). For each curve, the pre-operative mean angle was calculated, followed by the post-operative mean angle, and the percent correction was subsequently determined. All data have been presented as mean±standard deviation, where p<0.05 was considered statistically significant.

Surgical technique

The intra-operative neurophysiologic monitoring (IONM) and intra-operative fluoroscopy were used in all surgeries to assess screw placement. Pedicle screw placement was done using free hand technique using intra-operative visible and palpable anatomic landmarks for accurate screw insertion. Polyaxially pedicle screws were placed at key vertebrae to achieve coronal and sagittal correction, and the position of the pedicle screws was confirmed under fluoroscopy following their insertion to ensure accurate placement.

Adequate soft tissue and posterior ligament release was performed, with excision of the spinous processes, ligamentum flavum, laminae, and inferior articular facet joints. This step is crucial to be performed prior to reduction maneuvers, as it improves flexibility and ensures the safe reduction of the curve.

A first short pre-contoured rod is placed on the concave side of the major structural curve at the apical region to engage 4-6 pedicle screws. The short rod is then first fixed to the proximal and distal pedicle screws before engaging in between the peri-apical pedicle screws, creating a corrective translation. The apical region deformity was further corrected by rod rotation and distraction.

A second long pre-contoured rod in the ideal sagittal plane is placed to engage the rest of the pedicle screws on the concave side of the major structural curve. The long rod loosely connected to the pedicle screw and then the rod is rotated 90° in axial plane, thereby converting the coronal deformity into proper sagittal alignment. Segmental distraction along this long and short rod is done to further restore the global balance based on the Harrington stable zone. Sequential and gradual distraction over multiple vertebral segments is essential for better stress distribution.

Finally, a third long pre-contoured rod connected to the convex side of the curve to stabilize the construct. Sequential compression is done to further level the proximal thoracic and distal lumbar vertebrae to improve coronal and sagittal balance. It is important to monitor for signs of pedicle screw pullout during the reduction maneuver and to observe any changes in neuromonitoring throughout the reduction procedure.

The laminae and transverse processes are decorticated with an osteotome, and bone graft (autograft) is placed over the entire decorticated area. Crosslinks were placed between the long rods to control vertebral rotation and improve the stability of the construct.

## Results

A total of eight patients with severe rigid scoliosis underwent surgical correction using the triple-rod technique. All were females and their mean age at the time of surgery was 16.3 years (range: 13-24 years). The pre-operative mean major coronal Cobb angle was 97.9°±7.1° (mean±SD), which improved to a mean of 51.6°±10.9° post-operatively. The kyphotic angle improved from 53.9°±23.5° to 35.2°±9.5°. Our case series shows significant corrections across all curves, with thoracolumbar/lumbar (TL/L) curves achieving the highest correction rate (53%) and main thoracic (MT) showing the most statistically significant correction (Table 1). The technique achieved substantial correction across all curve types, with the main thoracic curve showing the greatest improvement, followed by the thoracolumbar/lumbar curves and proximal thoracic curves. There were no major intra-operative complications, such as neurological deficits or vascular injuries.

**Table 1:** Summary of radiographic outcomes. The proximal thoracic (PT) curve involves the upper thoracic spine from T1 to T5. The main thoracic (MT) curve is the primary and most significant curve extending from T5 to T12. The thoracolumbar/lumbar (TL/L) curve involved the lower thoracic and lumbar regions, spanning from T10 to L4. Kyphosis (K) is measured between the superior endplate of T5 and the inferior endplate of T12. All the Cobb angles were measured in degrees. Correction rate indicated the mean correction rates in percentage before the operation and after the operation. P-value means comparison before the operation and after the operation.

Curve type	Pre-operative mean (degrees)	Post-operative mean (degrees)	Correction rate (%)	p-Value
Proximal thoracic (PT)	23.0	15.1	34.3	<0.01
Main thoracic (MT)	97.9	51.6	47.3	<0.001
Thoracolumbar/lumbar (TL/L)	53.8	25.2	53.0	<0.01
Kyphosis (K)	53.9	35.2	34.6	<0.01

Table 2 illustrates the radiological outcomes following the triple-rod technique, demonstrating significant improvements in both coronal and sagittal plane deformities. Pre-operatively, the mean Cobb angle for the main thoracic (MT) curve was 97.88°, which reduced to 51.62° post-operatively, indicating a substantial correction. Similarly, the thoracolumbar/lumbar (TL/L) curve showed marked improvement, decreasing from 53.75° to 25.25°. The proximal thoracic (PT) curve, though less severe, also improved, with an average reduction from 23.02° to 15.12°. In the sagittal plane, kyphosis (K) was significantly corrected, with the pre-operative mean angle of 53.88°decreasing to 35.25° after surgery. Among the coronal deformities, the MT curve had the most significant correction, with an average reduction of 46.26° (47.3%), while kyphosis correction was 18.63° (34.6%).

**Table 2:** Demographic and radiological parameter. PT: proximal thoracic; MT: main thoracic; TL/L: thoracolumbar/lumbar; K: kyphosis; EOS: early onset scoliosis; AIS: adolescent idiopathic scoliosis; SS: syndromic scoliosis; NMS: neuromuscular scoliosis All the Cobb angles were measured in degrees.

Patient	Gender	Age (years)	Diagnosis	Pre-operative Cobb angle (degrees)				Post-operative Cobb angle (degrees)			
				PT	MT	TL/L	K	PT	MT	TL/L	K
1	F	13	EOS	17	98	74	74	15	58	35	45
2	F	19	AIS	18	92	79	9	14	36	22	22
3	F	14	SS	41	113	75	29	27	57	30	23
4	F	15	NMS	19	96	47	78	7	45	18	49
5	F	15	EOS	25	96	12	70	18	68	12	40
6	F	24	AIS	30	90	20	50	15	38	22	35
7	F	15	SS	17.2	93	70	50	15	45	36	35
8	F	15	AIS	17	105	53	71	10	66	27	33

**Figure 1:**
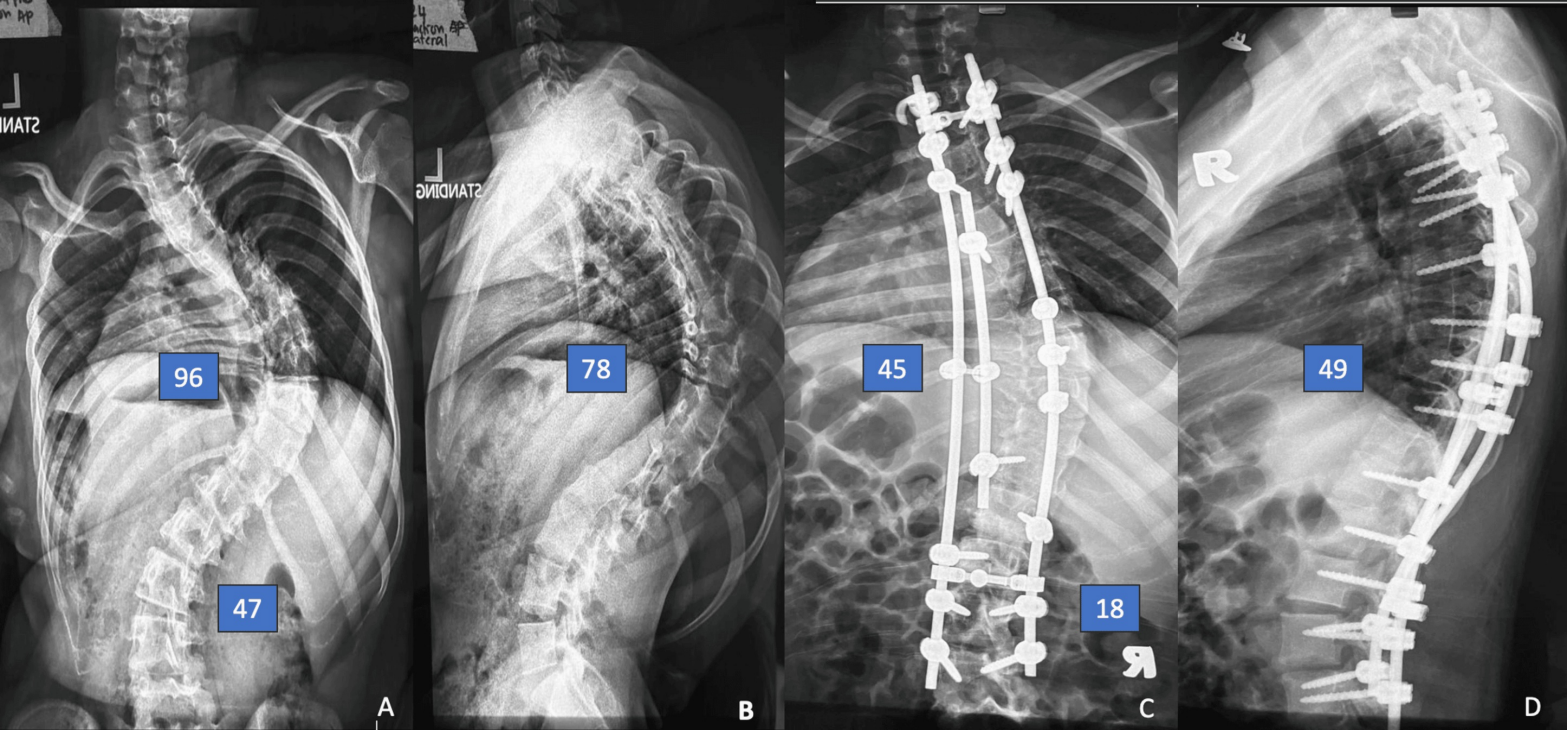
Whole spine radiograph of patient number 4. The pre-operative main thoracic (MT) Cobb angle of 96°, thoracolumbar/lumbar (TL/L) 47° (A) and thoracic kyphosis 78° (B). She underwent posterior instrumentation and fusion using triple-rod technique from T2-L4. The post-operative x-rays show coronal curve correction of MT to 45°, TL/L to 18° (C), and kyphosis correction to 49° (D). The details are provided in Table 2.

**Figure 2:**
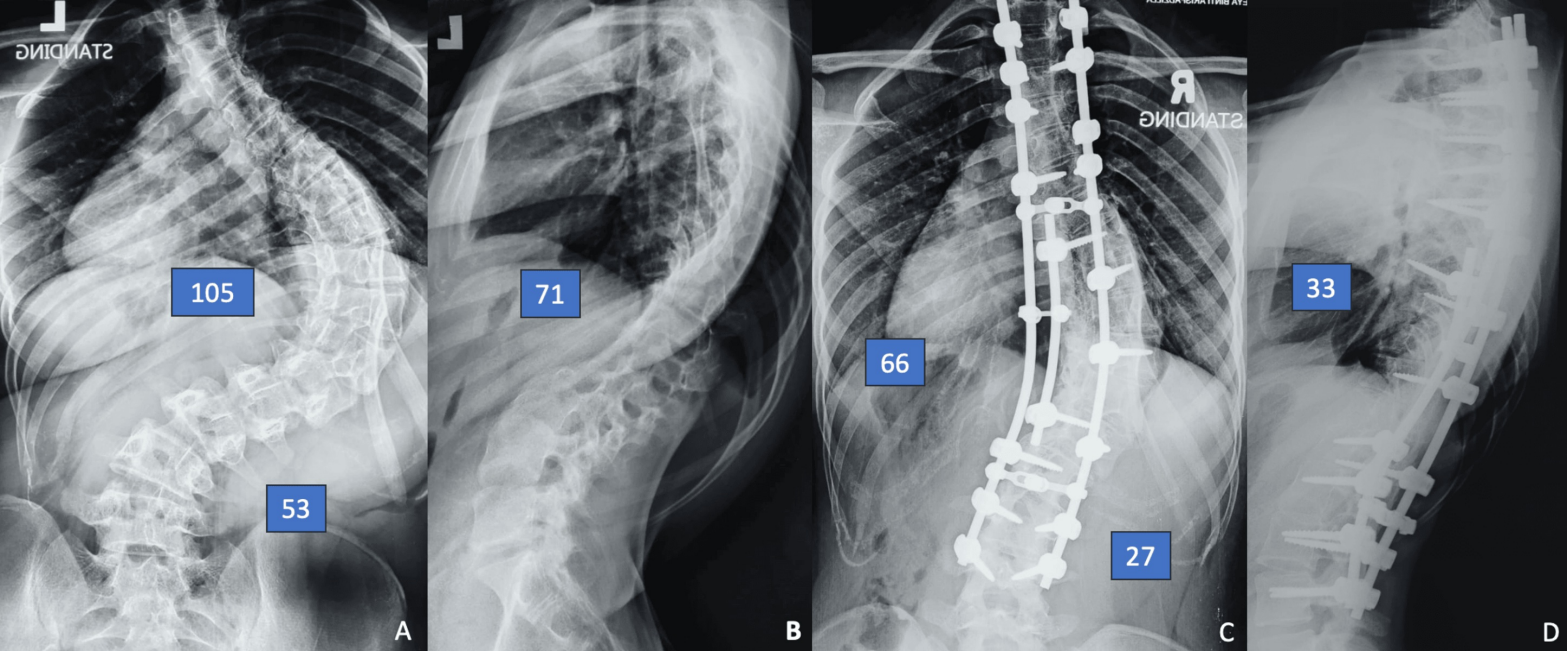
Whole spine radiograph of patient number 8. The pre-operative main thoracic (MT) Cobb angle of 105°, thoracolumbar/lumbar (TL/L) 53° (A) and thoracic kyphosis 71° (B). She underwent posterior instrumentation and fusion using triple-rod technique from T2-L4. The post-operative x-rays show coronal curve correction of MT to 66°, TL/L to 27° (C) and kyphosis correction to 33° (D). The details are provided in Table 2.

This approach has demonstrated effectiveness across various etiologies of rigid scoliosis, including adolescent idiopathic scoliosis (AIS), neuromuscular scoliosis (NMS), syndromic scoliosis (SS), and early-onset scoliosis (EOS). Figures 1A-1D illustrate pre- and post-operative whole spine images of a 15-year-old girl with underlying NMS, while Figures 2A-2D illustrate another 15-year-old girl with underlying AIS. The radiological outcomes following the triple-rod technique demonstrate significant improvements in both coronal and sagittal plane deformities. These findings indicate that the triple-rod technique is effective in achieving significant spinal correction in severe rigid scoliosis arising from various etiologies. The technique not only provided good alignment in the coronal plane but also improved sagittal balance, which is critical for long-term functional outcomes.

## Discussion

This case series have shown that the triple-rod technique is an effective and safe method for surgical handling of rigid scoliosis of the severe type. Severe rigid scoliosis poses significant surgical challenges, as these curves are often associated with greater degrees of rigidity, lack of flexibility for correction of the curve, and the presence of compensatory deformities that make correction even more difficult [5].

There are several anatomical variants, such as a significantly smaller or dysplastic pedicle on the concave side, deviation of the body towards the convex side, and a thicker pedicle on the convex side, commonly seen in scoliotic vertebrae [6,7]. These variants make pedicle screw insertion particularly challenging, especially in the apical region of the curve [3]. Critical medial wall perforation of the pedicle raises greater concern for the operating surgeon due to the risk of neurological complications. This is because the shifted neural structures towards the concave side of the curve cause proximity of the dura and medial cortex of the concave pedicle [8-10].

Conventionally, posterior osteotomies like pedicle subtraction osteotomy or vertebral column resection have been used to address rigid spinal deformities. However, these procedures are technically demanding, associated with higher morbidity, and can potentially compromise the stability of the spinal column [5,11]. Common complications associated with spine osteotomy are bleeding, neurological injury, infection, and nonunion or pseudoarthrosis [5,12]. Individuals with severe and rigid scoliosis typically exhibit significant thoracic deformities, frequently accompanied by cardiopulmonary impairment. These patients may not be suitable candidates for vertebral column resection due to the prolonged surgical duration and substantial blood loss, nor can they withstand the risk of pulmonary complications associated with anterior surgical approaches [13,14].

An alternative to osteotomy is pre-operative halo gravity traction for a period of four to eight weeks. This technique serves as a gradual adjunct to the primary treatment, rather than offering definitive deformity correction [15,16]. The existing literature on the efficacy of pre-operative traction is mixed. While some studies, such as by Sponseller et al., have suggested that the technique does not enhance surgical correction, others, like the study by Rinella et al., have reported a 46% improvement in 33 patients treated for severe scoliosis [16,17].

We perform limited multiple osteotomies or wide inferior facetectomies at all levels of the intended fusion. This surgical technique offers several benefits, including improved spinal mobility, enhanced visualization of anatomical landmarks for pedicle screw placement, provision of bone graft, and exposure of more bone surface for fusion, all of which further enhance the posterior fusion [18]. The multilevel osteotomies facilitate a gradual restoration of the spinal curvature across multiple vertebral levels while also distributing the stress evenly among the osteotomy sites, thereby mitigating the risk of excessive structural compromise [19].

The triple-rod technique offers a safe and effective option for surgical treatment for severe rigid scoliosis. It allows for a controlled and gradual correction of apical region deformity followed by global spinal realignment [13,20]. This sequential correction of deformity significantly minimizes the risk of neurological complications and allows substantial correction of the coronal and sagittal deformities. The application of the first short pre-contoured rod at the concave side apical region allows a reduction in coronal deformity of the major structural curve. The short rod on the concave side applies strong tension, targeting the rigid apex of the scoliosis. Apical region correction reduces the need for extensive pre-bending of the long rods. This is because rotating and distracting via the short rod has partially corrected the most rigid area, reducing the overall Cobb angle for deformity. This approach minimizes the risk of pedicle screw pullout and simplifies sagittal plane reconstruction by requiring less pre-bending of the long rods [18,20,21].

In this case series for severe and rigid scoliosis (major curves >90°), the surgical strategy of apical region correction and global balance with three rods provided major curve coronal plane correction up to 53%. As for the mean average of spinal sagittal kyphosis angle, it is 35.2±9.5 degrees. Post-operative clinical evaluation shows that both coronal and sagittal balance were improved and maintained for at least one year. The technique achieved substantial correction across all curve types, with the thoracolumbar/lumbar curve showing the greatest improvement, followed by the main thoracic curves and proximal thoracic curves. The findings from this case series are consistent with the published literature regarding the use of the triple-rod technique for managing severe and rigid scoliosis [13,20].

In comparison to complex osteotomy techniques, such as vertebral column resection, pedicle subtraction osteotomy, or anterior release, for managing severe spinal deformities, the triple-rod approach focusing on apical region correction and global balance has exhibited several benefits. The triple-rod technique has been linked to reduced operative duration, decreased blood loss, lower likelihood of neurological complications, and fewer occurrences of nonunion or pseudarthrosis [5,13,18,22,23]. In our limited number of cases, there is no case of surgical complications such as neurological deficit, infection, or pseudarthrosis.

This study has several limitations. The small sample size and the heterogeneity of etiology may affect the generalizability of the findings. Further research is required to better characterize the surgical outcomes in patients with different underlying conditions. Additionally, the lack of long-term follow-up limits the ability to assess the durability of the correction, potential late-onset complications, and overall functional outcomes. In addition, the study was limited by all the shortcomings associated with a retrospective study.

## Conclusions

Surgical treatment of severe rigid scoliosis poses significant challenges for the operating surgeon. The triple-rod technique provides an effective option for correcting severe rigid scoliosis, offering substantial correction of coronal and sagittal deformities arising from various underlying etiologies. A key advantage of this technique is its ability to achieve a gradual and controlled correction of spinal deformity. By allowing corrective forces to be distributed sequentially and evenly across multiple levels, it facilitates a more stable restoration of spinal alignment. Additionally, it helps reduce the risk of neurological complications and other surgical morbidities associated with severe rigid scoliosis surgery.
